# The Development of the Adaptive Behavior Scale for Stroke Survivors

**DOI:** 10.3390/healthcare12171719

**Published:** 2024-08-29

**Authors:** Hyunsuk Choi, Youngshin Song

**Affiliations:** 1 Department of Nursing, Cheongju University, Cheongju 28503, Republic of Korea; ilsu7729@gmail.com; 2 Department of Nursing, Chungnam National University, Daejeon 35015, Republic of Korea

**Keywords:** adaptation, factor analysis, psychometrics, stroke

## Abstract

The adjustment of stroke survivors is self-directed and multi-dimensional. This study developed an adaptive behavior scale for stroke survivors reflecting these characteristics and performed a psychometric evaluation. The item pool was derived based on conceptual attributes and indicators of adaptive behaviors for stroke survivors. Ten experts assessed the content validity. The scale was refined through pilot testing and interviews with 10 stroke survivors. From December 2021 to May 2022, a self-report questionnaire consisting of a five-point Likert scale was administered to 215 stroke survivors visiting a university hospital in S City, South Korea. Item analysis and confirmatory factor analysis were conducted to assess the construct validity; reliability was confirmed using Cronbach’s α. The final scale comprised three factors and 16 items: taking an optimistic view, restructuring daily activities to suit oneself, and carrying out one’s daily life. The confirmatory factor analysis indicated a good fit for the three-factor model; Cronbach’s α coefficient of the scale was 0.90, indicating a very good internal consistency. This easy-to-use, concise self-report scale applies to stroke survivors from subacute to chronic stages, providing healthcare professionals with the basic data needed to assess their adaptation. It may also facilitate individualized intervention program development to improve stroke survivor adaptation.

## 1. Introduction

Worldwide, stroke is the second and the third leading cause of death and disability-adjusted life years, respectively [1]. A stroke has a huge impact on an individual’s life, even if they survive, because it comes on suddenly and causes various changes [2]. Stroke survivors experience limitations in their bodily functions [3], changing roles at home and in society [4], unemployment and economic hardship [5], and negative emotions such as depression and anger [6].

Adaptation to chronic illness is defined as a comprehensive physiological and psychological factor that responds to internal and external stimuli related to the illness [7]. Chronic illness self-management includes not only the assessment of changes in physical signs and symptoms, but also responses to changes, perception of situations, emotional symptoms, and goal-setting [8]. Regarding the adaptation of stroke survivors, recovery of physical functions such as motor function and the activities of daily living has been given priority, but recent research results have reported that psychological and social factors such as self-efficacy, resilience, and social support have a significant impact on adaptation after a stroke [9,10,11]. Therefore, a multi-dimensional approach that includes psychological, social, and physical aspects is required to help stroke survivors adapt [6]. In order to prioritize interventions to improve stroke survivors’ adaptation in practice, scales that can assess their adaptation from multiple perspectives and provide objective information are needed.

There are existing scales to assess the status of stroke survivors. The Stroke-Specific Quality of Life Scale (SS-QoL) [12], Stroke Impact Scale (SIS) 3.0 [13], Preference-Based Stroke Index (PBSI) [14], and Post-Stroke Checklist (PSC) [15] are representative examples. Although these tools contribute to providing information about stroke survivors, they are mainly focused on measuring clinical outcomes such as symptoms and functions. In addition, since the number of items is large or since it was developed for survivors in the early stages of stroke, there are restrictions in applying them to survivors with various time points and symptoms. Therefore, a concise self-report scale that can assess the adaptation of various stroke survivors would be useful in practice.

In the case of congenitally disabled, including intellectual and developmental disabilities, adaptive behaviors are evaluated to set goals for education and rehabilitation [16]. Adaptive behavior is an intentionally learned action defined as the level of daily performance of tasks necessary to fulfill normal roles in society, including maintaining independence and meeting the cultural expectations of personal and social responsibility [17]. However, the adaptive behavior of stroke survivors is a self-directed process characterized by understanding, acceptance, compromise, and adjustment to sudden changes due to the disease, and it is different from that of individuals with congenital disabilities [18]. Therefore, the adaptive behavior scale for stroke survivors (ABS-SS), which reflects the conceptual properties and components of the adaptive behavior of stroke survivors, will provide meaningful information about them.

The present study aimed to develop and validate the ABS-SS, which can be comprehensively applied to stroke survivors at various times and conditions and can assess psychological, social, and physical aspects.

## 2. Materials and Methods

### 2.1. Study Design

This was a methodological study to develop the ABS-SS using a cross-sectional design.

### 2.2. Procedures

This study was based on the scale development procedure described by Netemeyer et al. [19].

#### 2.2.1. Construct Definition and Content Domain

The construct definition and content domains of the concept of adaptive behavior for stroke survivors were identified in a concept analysis study by Choi et al. [18].

#### 2.2.2. Generating and Judging Measurement Items

- Generating an item pool

The researchers stated the items in sentences, paraphrasing the indicators of the concept of adaptive behavior for stroke survivors so that the statements of each item reflect the structure of the concept. The items related to the representative symptoms experienced by stroke survivors were described in conditional sentences. Dysphagia, fatigue, pain, and hemiplegia, which were all included, are representative problems encountered by stroke survivors [20,21]; however, not all survivors experience them. In addition, as many items as possible were derived to identify the items that could test the concept of the intended instruction.

- Content validity

In order to evaluate the content validity, an expert panel was formed, including three nursing professors with extensive experience in scale development, two doctors of rehabilitation medicine, and five nurses with more than 10 years of experience in nursing stroke patients. This sample size met the criterion that 3–10 experts were adequate [22]. A content validity evaluation was conducted from 23 September to 6 October 2021. The expert panel rated the extent to which each item represented or was related to the concept of adaptive behavior for stroke survivors on a four-point Likert scale ranging from one (not relevant) to four (highly relevant). Additional opinions from the experts were also collected. The content validity confirmed that the item-level content value index (I-CVI) was 0.78 or higher, and the scale-level content validity index (S-CVI) was 0.90 or higher [23].

#### 2.2.3. Designing and Conducting Studies to Develop and Refine the Scale

To confirm the suitability and clarity of the items constituting the scale, a convenience sample was obtained from ten stroke survivors among the outpatients visiting the cardiocerebrovascular center of a university hospital located in S City, South Korea. Pilot testing and interviews were conducted from 18 to 26 November 2021. Participants completed a structured self-report questionnaire rated on a five-point Likert scale. The researchers had 10 respondents say what they thought when reading the items or explain what the items meant in their own words. They were also asked to make suggestions that could express the items more clearly. We considered the view that the validity can be improved when including the opinions of the target population [24].

#### 2.2.4. Finalizing the Scale

- Participants

In the psychometric evaluation phase, the participants were recruited using convenience sampling, targeting the outpatients of a university hospital’s cardiocerebrovascular center in S City, South Korea. The inclusion criteria were as follows: (1) people diagnosed with a stroke, (2) those aged 19 years or older, (3) the ability to understand the meaning of the questions, and (4) the ability to express one’s opinion. In total, 215 stroke survivors who voluntarily agreed to participate were included in this study. This sample size met the criterion that 150–400 samples would be desirable for a confirmatory factor analysis (CFA) based on the maximum likelihood estimation (MLE) [25].

- Data collection

The data collection was conducted face-to-face by a researcher from 7 December 2021 to 10 May 2022. The stroke survivors completed a structured self-report questionnaire comprising 36 items rated on a five-point Likert scale, 12 for participants’ general characteristics, and 5 for the EuroQol Five Dimensions (EQ-5D-5L) for the concurrent validity test. Before using the Korean EQ-5D-5L, we registered a research plan with the EuroQol Research Foundation and received approval for its use (Registration ID 42119). During data collection, if it was difficult for the participants to fill out the questionnaire alone because of hemiplegia, poor eyesight, or the inability to read, the researcher read the questions and checked their answers.

- Data analysis

The construct validity was assessed using an item analysis and a CFA. Prior to the item analysis, the bias of the items was reviewed by checking that the absolute values of skewness and kurtosis did not exceed two and seven, respectively [26]. Only items with an item-to-total correlation coefficient between 0.50 and 0.80 were retained by performing an item analysis [27]. In order to conduct CFA, researchers must propose a measurement model that specifies the number of factors and variables based on a theoretical review [25]. In this study, a primary measurement model was developed based on a concept analysis study [18], item analysis, and the opinions of an expert panel. The CFA was performed based on the MLE, and the goodness of fit of the factor structure was evaluated by applying the following indices and criteria: normed χ^2^ (CMIN/DF) ≤ 3.0, goodness of fit index (GFI) ≥ 0.90, adjusted GFI (AGFI) ≤ 0.85, standardized root mean residual (SRMR) ≤ 0.10 [28], root mean square error of approximation (RMSEA) ≤ 0.10 [29], comparative fit index (CFI) ≥ 0.90 [19], and Tucker–Lewis index (TLI) ≥ 0.90 [30]. The convergent validity was evaluated based on a standardized item-to-factor loading of 0.50–0.95 [31], average variance extracted estimate (AVE) ≥ 0.50, and composite reliability (CR) ≥ 0.70 [25]. The correlation coefficients between the factors were checked to assess the discriminant validity [32]. The concurrent validity was evaluated as appropriate if the correlation coefficient was in the range of 0.30–0.60 [33] by confirming the Pearson correlation between the ABS-SS and EQ-5D-5L. The reliability confirmed internal consistency, which is the degree to which the items in the scale measure the same attributes as Cronbach’s α coefficient [34]. Cronbach’s α coefficient was evaluated based on the view that 0.70–0.80 is ‘respectable’, 0.80–0.90 is ‘very good’, and 0.90 or higher indicates that the number of items should be reduced [35].

As there were no missing data, all the collected data were included in the analysis and assessed using IBM SPSS Statistics 26 and IBM SPSS AMOS 26 programs.

### 2.3. Ethical Considerations

The study was conducted in accordance with the Declaration of Helsinki, and approved by the Institutional Review Board of Chungnam National University (no. 202105-SB-071-01 and date of approval 3 September 2021). In all procedures of the study, the purpose of the study, its participants and methods, and the expected risks and benefits of participation were explained to the subjects. In addition, they were informed of the freedom to participate and withdraw from the study and the confidentiality of personal information. Informed consent was obtained from subjects who understood these conditions and voluntarily agreed to participate.

## 3. Results

### 3.1. Construct Definition and Content Domain

The concept of adaptive behavior for stroke survivors was identified using four attributes (realizing change, taking an optimistic view, restructuring daily activities to suit oneself, and carrying out one’s daily life) and 21 indicators [18].

### 3.2. Generating and Judging Measurement Items

- Generating an item pool

The researchers generated an item pool of 56 items that could represent the attributes and indicators of the concept to be measured. Specifically, six items were derived from two indicators of the first attribute, ‘realizing change’, 14 items from four indicators of the second attribute, ‘taking an optimistic view’, 24 items from 11 indicators of the third attribute, ‘restructuring daily activities to suit oneself’, and 12 items were derived from four indicators of the fourth attribute, ‘carrying out one’s daily life.’ This satisfied the view that the number of initial items should be about twice the number of final items [35].

- Content validity

In the content validity assessment by the expert panel, the I-CVI range of 55 items was 0.80–1.00, which met the criteria, and the S-CVI/Ave was 0.93. One item was evaluated as having a low correlation with the concept that the scale was intended to measure, so it was decided that it should be removed. In addition, by reflecting on the opinions of the experts, 10 items with low relevance or overlap were removed. Based on the review results of the expert panel, the scale was revised to contain a total of 45 items, and the response categories of the scale were organized into a five-point Likert scale to provide a stepwise difference in the possible responses.

### 3.3. Designing and Conducting Studies to Develop and Refine the Scale

The time taken to complete the questionnaire in the pilot test was 5–16 min. In the interviews, there were 11 items that several respondents answered as being difficult to respond to because the intention of the question was ambiguous or redundant or that it was difficult to respond to. There was no corresponding item. The researchers reviewed these items, removed nine items, and modified two. The preliminary ABS-SS consisted of a total of 36 items.

### 3.4. Finalizing the Scale

#### 3.4.1. Participants Characteristics

The mean age of the participants was 68.75 ± 11.27 years, and 66.5% were male. Among the participants, 31.2% had only graduated from elementary school, 64.2% were not employed, and 56.3% had a religion. The mean duration of stroke prevalence was 46.20 ± 60.75 months, and 35.3% exhibited hemiplegia. Table 1 presents the participants’ characteristics.

#### 3.4.2. Construct and Concurrent Validity

The mean score range of each item in the ABS-SS was 3.34–4.36; moreover, 36 items were included in the analysis because the skewness and kurtosis values of all items met the criteria. In the item analysis, 18 items were removed, considering the corrected item-to-total correlation coefficients. In this process, all items included in the ‘realizing change’ attribute were removed. Therefore, the first model was composed of three factors (taking an optimistic view, restructuring daily activities to suit oneself, and carrying out one’s daily life) and 18 items, and CFA was performed. The first model did not have a good fit: CMIN/DF = 3.18, GFI = 0.81, AGFI = 0.75, SRMR = 0.05, RMSEA = 0.10, CFI = 0.84, and TLI = 0.82. Thus, we focused on three items (A11, A20, and A28) with the item-to-factor loadings of 0.50 or less. A11 “I am learning how to live together while managing my stroke” was deleted. A28 was moved because it was judged to be more suitable for the factor of ‘taking an optimistic view’; it was decided to keep A20. Additionally, A19 “I am still a person of value” was removed because it had a similar meaning to A24 “I am still living a meaningful life.” A35 was also judged to be a more suitable item for the factor of ‘taking an optimistic view’; hence, the factor was moved. Finally, CFA was performed with a modified model with three factors and 16 items. The second model fit indices were CMIN/DF = 2.39, GFI = 0.87, AGFI = 0.82, SRMR = 0.05, RMSEA = 0.08, CFI = 0.91, and TLI = 0.89, which were confirmed as acceptable. In the second model, the range of standardized factor loadings of 16 items was 0.47–0.91, which was acceptable. Since the C.R. was always 1.96 or higher, it was confirmed to be statistically significant (Table 2).

**Figure 1:**
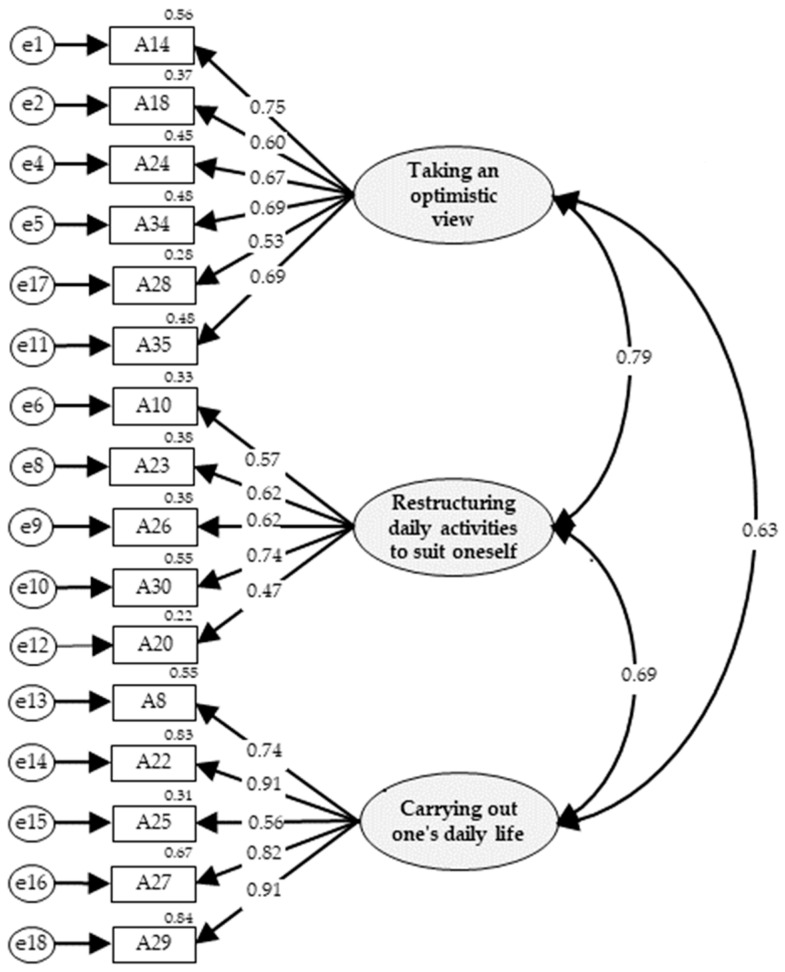
Final model of the adaptive behavior scale for stroke survivors.

The AVE values of the three factors were 0.60, 0.60, and 0.72, respectively, and the CR values were 0.90, 0.88, and 0.92, respectively, which were appropriate for the convergent validity evaluation criteria. The discriminant validity confirmed that the confidence intervals of the correlation coefficient between factors were 0.74–0.84, 0.58–0.68, and 0.63–0.75, respectively, and this did not include 1. Each factor can be evaluated as discriminatory (Table 3). Therefore, the second model was preferred as the final model (Figure 1). There was a significant positive correlation between the total ABS-SS score and EQ-5D-5L score (r = 0.48, *p* < 0.010), confirming the concurrent validity of the ABS-SS. The Pearson correlation between ABS-SS’ sub-factors (taking an optimistic view, restructuring daily activities to suit oneself, and carrying out daily life) and EQ-5D-5L was 0.49, 0.16, and 0.50, respectively, which were all statistically significant.

#### 3.4.3. Reliability

The overall Cronbach’s α coefficient for the ABS-SS was 0.90, indicating a very good internal consistency despite the small number of items. The Cronbach’s α coefficients for the three factors (taking an optimistic view, restructuring daily activities to suit oneself, and carrying out daily life) were identified as 0.82, 0.74, and 0.89, respectively, thus signifying a good internal consistency (Table 4).

## 4. Discussion

The ABS-SS developed in this study reflects the attributes of the stroke survivors’ adaptive behavior; furthermore, its validity and reliability were evaluated as good. The factor ‘taking an optimistic view’ (six items) can measure psychological aspects such as hope, self-efficacy, self-esteem, and emotional stability. The factor ‘restructuring daily activities to suit oneself’ (five items) can measure adjustments and compromises related to symptoms frequently experienced by stroke survivors such as food intake, fatigue and pain management, and use of assistive devices and resources. Lastly, the ‘carrying out one’s own daily life’ factor (five items) included activities of daily life such as eating, changing clothes, bathing, urination/defecation, and movement (see Appendix A). The ABS-SS, which includes multi-dimensional aspects, is useful for understanding the process of adaptation to the physical, social, and psychological changes after the acute phase of a stroke. Therefore, it can provide useful information to healthcare professionals who care for stroke survivors not only in acute hospitals but also in the community.

Initially, the items of the ABS-SS were composed of four factors based on the concept analysis of Choi et al. [18], but during the psychometric evaluation process, the ‘realizing change’ factor was deleted, so the final scale preferred three factors. The removed factor consisted of questions about having changed physically, socially, and psychologically due to a stroke and living by accepting these changes. The items included in this factor had a low corrected item-total correlation value. We interpreted this result as related to the fact that the data collection site of this study was an outpatient clinic. The participants were discharged after experiencing a critical situation, and they visited the outpatient clinic regularly because they were aware of and accepted the changes after the stroke. Therefore, future research that expands the scope of data collection is needed.

In the item reduction process, redundancies with similar items and low values in the corrected item-total correlations were excluded; however, not all items with low values were removed. One item (A20), “*If it is difficult to walk alone due to a stroke, I will use an assistive device such as a cane, crutches, or a wheelchair*” was included despite the low estimated value. As hemiplegia is the most common problem experienced by stroke survivors [36], this question was confirmed to be highly related to the concept measured by experts. Therefore, we decided to retain this item in the ABS-SS to reflect the characteristics of stroke survivors. 

Factor analysis is a popular and appropriate method for assessing the dimensionality of a construct. While exploratory factor analysis (EFA) is used to gain insight into the latent dimensionality of items and scales, CFA focuses on whether the hypothesized factor model fits the data [19]. Since we were able to specify the construct factors through concept analysis by Choi et al. [18] and item analysis, we attempted to confirm the dimensionality using CFA. In the CFA results of the second model consisting of three factors and 16 items, most fit indices met the criteria. Considering these statistical criteria and the conceptual meaning of the items, we decided that the second model was the final model.

In order to confirm the concurrent validity of ABS-SS, the correlation with EQ-5D-5L, a health-related quality of life measurement index, was confirmed. Since EQ-5D-5L is a valid tool for measuring the general health status of stroke survivors [37], it was judged to be suitable as a criterion tool for confirming the concurrent validity of the developed tool. The total score of the ABS-SS and the EQ-5D-5L showed a moderate significant correlation, so the concurrent validity was secured. Specifically, the ‘taking an optimistic view’ and ‘carrying out one’s own daily life’ factors of the developed tool and the EQ-5D-5L showed a moderate correlation. However, the ‘restructuring daily activities to suit oneself’ factor showed a low correlation. These results are because the EQ-5D-5L consists of items to measure mobility, self-care, usual activities, pain/discomfort, and anxiety/depression, and is far from the items that constitute the factor of ‘restructuring daily activities to suit oneself’. This is the best strength of the ABS-SS. Previous scales focus on the clinical outcome and status of stroke survivors’ adaptation. However, ABS-SS includes awareness of the process of self-directed adaptation and the need to compromise with the changes caused by stroke. This is a distinctive characteristic of the adaptive behavior of stroke survivors, who experience a major change in their lives due to a sudden stroke, unlike those with congenital disabilities. This is similar to the report by Lawless et al. [38] that self-management of long-term conditions (LTC) by older adults is a dynamic and continuous process of future-oriented behavioral adaptation to address LTC-related stressors and maintain a positive quality of life.

Another strength of the ABS-SS is that it is concise and easily structured, allowing for self-reporting, and this will be useful in increasing the response rate. In particular, we expect that reflection and individual goal-setting will occur during the self-reporting process. The ABS-SS can be used as a basis for healthcare professionals caring for stroke survivors to objectively evaluate stroke survivors’ adaptive behaviors and to understand adaptation at various times and in various situations. Furthermore, it may contribute to the development of individualized intervention programs in which stroke survivors can improve adaptive behavior.

Although the ABS-SS is valid and reliable, this study had some limitations. First, the data were collected from one university hospital using convenience sampling. The results must be interpreted carefully, and their generalizability is limited. Second, the disease duration and severity were not considered as the subject selection criteria. Third, EFA was not performed. We did not have a rigorous test of which items belonged to which factors or which factors were loaded. Finally, the expert panel’s composition may introduce bias, as it was composed primarily of nursing and rehabilitation medicine backgrounds. Therefore, further studies reflecting the opinions of multidisciplinary experts are needed.

Based on the present study, we suggest further studies on larger populations to confirm the validity of the theoretical structure of the ABS-SS. Additionally, longitudinal studies are required to evaluate the sensitivity of the scale and the predictive validity for long-term outcomes.

## 5. Conclusions

ABS-SS was developed according to the scale development procedure. The theoretical structure of stroke survivors’ adaptive behavior was confirmed, and an appropriate three-factor model and 16 items were constructed in consideration of the conceptual meaning and statistical criteria of the items. The validity of ABS-SS was confirmed for stroke survivors, and despite the small number of items the reliability was confirmed to be very good, so it will be useful for clinical application.

## Figures and Tables

**Table 1:** General characteristics of the participants (*n* = 215).

Characteristics	Categories	*n* (%)	M ± SD (min–max)
Sex	Men	143 (66.5)	
	Women	72 (33.5)	
Age (years)			68.75 ± 11.27 (27–90)
	≤54	21 (9.8)	
	55–64	45 (20.9)	
	65–74	83 (38.6)	
	75–84	54 (25.1)	
	≥85	12 (5.6)	
Education level	Elementary school	67 (31.2)	
	Middle school	37 (17.2)	
	High school	65 (30.2)	
	College	46 (21.4)	
Employed	Yes	77 (35.8)	
	No	138 (64.2)	
Have a religion	Yes	121 (56.3)	
	No	94 (43.7)	
Perceived health status	Very good	7 (3.3)	
	Good	52 (24.2)	
	Fair	95 (44.2)	
	Poor	59 (27.4)	
	Very poor	2 (0.9)	
Perceived economic satisfaction	Very good	3 (1.4)	
	Good	69 (32.1)	
	Fair	107 (49.8)	
	Poor	32 (14.9)	
	Very poor	4 (1.9)	
Duration of stroke (months)			46.20 ± 60.75 (0–360)
	≤6	58 (27.0)	
	7–24	60 (27.9)	
	≥25	97 (45.1)	
Hemiplegia	Yes	76 (35.3)	
	Left	39 (18.1)	
	Right	37 (17.2)	
	No	139 (64.7)	
With a caregiver	Yes	179 (83.3)	
	Spouse	109 (50.7)	
	Parents	6 (2.8)	
	Children	48 (22.3)	
	Relative	10 (4.7)	
	Facility	6 (2.8)	
	No	36 (16.7)	

M = mean; SD = standardized deviation.

**Table 2:** Comparison of loading estimates from the first and final models (*n* = 215).

Items	First Model				Second Model				Revised
	Standardized Estimates	S.E.	SMC	C.R.	Standardized Estimates	S.E.	SMC	C.R.	
***Factor 1. Taking an optimistic view***									
A14	0.74		0.55		0.75		0.56		
A18	0.63	0.14	0.39	8.73	0.60	0.14	0.37	8.52	
A19	0.68	0.13	0.47	9.54					Deleted
A24	0.75	0.13	0.57	10.50	0.67	0.13	0.45	9.41	
A34	0.63	0.10	0.39	8.72	0.69	0.10	0.48	9.70	
A28					0.53	0.13	0.28	7.34	
A35					0.69	0.10	0.48	9.75	
***Factor 2. Restructuring daily activities to suit oneself***									
A10	0.55		0.30		0.57		0.33		
A11	0.45	0.19	0.20	5.44					Deleted
A23	0.57	0.16	0.32	6.44	0.62	0.16	0.38	6.54	
A26	0.63	0.11	0.40	6.93	0.62	0.10	0.38	6.85	
A30	0.70	0.11	0.49	7.34	0.74	0.11	0.55	7.48	
A35	0.60	0.14	0.37	6.72					Modified to factor 1
A20	0.44	0.14	0.19	5.36	0.47	0.14	0.22	5.62	
***Factor 3. Carrying out one’s daily life***									
A8	0.75		0.56		0.74		0.55		
A22	0.91	0.06	0.83	14.23	0.91	0.06	0.83	14.03	
A25	0.56	0.11	0.32	8.31	0.56	0.11	0.31	8.20	
A27	0.81	0.05	0.67	12.57	0.82	0.05	0.67	12.45	
A28	0.40	0.08	0.16	5.86					Modified to factor 1
A29	0.91	0.05	0.83	14.25	0.91	0.05	0.84	14.07	

S.E. = standardized error; SMC = squared multiple correlation; C.R. = critical ratio.

**Table 3:** Convergent validity and discriminant validity (*n* = 215).

Factors	Correlation Coefficients ± 2 × S.E			AVE	CR
	1	2	3		
1. Taking an optimistic view				0.60	0.90
2. Restructuring daily activities to suit oneself	0.74–0.84			0.60	0.88
3. Carrying out one’s daily life	0.58–0.68	0.63–0.75		0.72	0.92

AVE = average variance extracted estimate; CR = composite or construct reliability.

**Table 4:** Reliability (*n* = 215).

Factors	Number of Items	Cronbach’s α
Taking an optimistic view	6	0.82
Restructuring daily activities to suit oneself	5	0.74
Carrying out one’s daily life	5	0.89
Total	16	0.90

## Data Availability

Data are contained within the article.

## Appendix A

**Table A1:** The adaptive behavior scale for stroke survivors (ABS-SS).

Factors	No	Items	Strongly Agree (5)	Agree (4)	Neutral (3)	Disagree (2)	Strongly Disagree (1)
Taking an optimistic view	1	I have hope that my condition will be better than now					
	2	I don’t despair of having a stroke					
	3	I can endure it even if the rehabilitation period is long					
	4	I am living a meaningful life					
	5	I have a role that suits me at home and in society					
	6	I can calm myself down when my mind is unstable					
Restructuring daily activities to suit oneself	7	If I have difficulty swallowing food due to a stroke, I will switch to easier-to-eat foods, such as porridge or liquid food					
	8	If the stroke causes fatigue, I will control my activities so that I don’t overwork myself					
	9	If I have pain from a stroke, I will find ways to reduce the pain, such as medication, physical therapy, and rest					
	10	If I need help, I can ask for help from family or relatives					
	11	If it is difficult to walk alone due to a stroke, I will use an assistive device such as a cane, crutches, or a wheelchair					
Carrying out daily life	12	I eat myself					
	13	I dress and undress by myself					
	14	I wash myself					
	15	I go to the toilet by myself					
	16	I drive or use public transportation or an accessible vehicle to get out of town					
